# Idiopathic (primary) achalasia: a review

**DOI:** 10.1186/s13023-015-0302-1

**Published:** 2015-07-22

**Authors:** Dhyanesh A. Patel, Hannah P. Kim, Jerry S. Zifodya, Michael F. Vaezi

**Affiliations:** Department of Internal Medicine, Nashville, TN USA; Division of Gastroenterology, Hepatology and Nutrition, Vanderbilt University Medical Center, Nashville, TN USA

**Keywords:** Esophagus, Achalasia, Motility disorder, Endoscopic balloon dilatation, Laparoscopic surgery

## Abstract

Idiopathic achalasia is a primary esophageal motor disorder characterized by loss of esophageal peristalsis and insufficient lower esophageal sphincter relaxation in response to deglutition. Patients with achalasia commonly complain of dysphagia to solids and liquids, bland regurgitation often unresponsive to an adequate trial of proton pump inhibitor, and chest pain. Weight loss is present in many, but not all patients. Although the precise etiology is unknown, it is often thought to be either autoimmune, viral immune, or neurodegenerative. The diagnosis is based on history of the disease, barium esophagogram, and esophageal motility testing. Endoscopic assessment of the gastroesophageal junction and gastric cardia is necessary to rule out malignancy. Newer diagnostic modalities such as high resolution manometry help in predicting treatment response in achalasia based on esophageal pressure topography patterns identifying three phenotypes of achalasia (I-III) and outcome studies suggest better treatment response with types I and II compared to type III. Although achalasia cannot be permanently cured, excellent outcomes are achieved in over 90 % of patients. Current medical and surgical therapeutic options (pneumatic dilation, endoscopic and surgical myotomy, and pharmacologic agents) aim at reducing the LES pressure and facilitating esophageal emptying by gravity and hydrostatic pressure of retained food and liquids. Either graded pneumatic dilatation or laparoscopic surgical myotomy with a partial fundoplication are recommended as initial therapy guided by patient age, gender, preference, and local institutional expertise. The prognosis in achalasia patients is excellent. Most patients who are appropriately treated have a normal life expectancy but the disease does recur and the patient may need intermittent treatment.

## Definition and epidemiology

Idiopathic achalasia (ORPHA930) is a primary esophageal motility disorder of unknown etiology characterized manometrically by esophageal aperistalsis and insufficient relaxation of the lower esophageal sphincter (LES) in response to deglutition [1–5]. It is a rare disease with an annual incidence of approximately 2/100,000 and a prevalence rate of 10/100,000 [6]. Studies have shown that incidence and prevalence of the disease are increasing [7, 8] and the peak incidence occurs between 30 and 60 years of age [7]. Achalasia was first described and termed by Sir Thomas Willis in 1674, when he suggested that the disease is due to the loss of normal inhibition in the distal esophagus [9]. Since then, the development of new diagnostic techniques stimulated new ideas about the etiology and pathophysiology of the disease leading to various theories in identifying the nature of motor disturbances in esophageal regions. However, the initiating cause is still unclear [3, 9, 10].

In this review article we provide current insight on the pathogenesis, etiology, diagnosis, and treatment options for this motor disorder of the esophagus.

## Clinical description

Achalasia is one of the most investigated motor disorders of the esophagus [4, 10]. The disease can occur at any age but it is usually diagnosed between 30 and 60 years. Progressive dysphagia to solids followed by liquids (82 %-100 %) is the first clinical symptom of achalasia [11]. Although dysphagia can occur in patients with other esophageal motility disorders, this symptom is most characteristic of achalasia and strongly suggests the diagnosis.

Regurgitation not responding to adequate proton pump inhibitor (PPI) therapy and weight loss can be seen in 30 % to 90 % of patients. Regurgitation of material retained in the dilated esophagus, especially during supine position at night, may lead to aspiration. There is no universal definition for “adequate” PPI therapy, however, based on recent gastro-esophageal reflux disease (GERD) guidelines, it has been taken to constitute ensuring compliance, optimal dosing, changing to a different PPI, and possibly BID dosing [12]. Chest pain is another presenting symptom of achalasia (17 %-95 %). The occurrence of this symptom is unrelated to the LES pressure [4, 11]. Chest pain has been found to be more frequent in younger patients and in female patients who have achalasia [4, 13–15]. In addition, 40 % of patients with achalasia report occurrence of at least one respiratory symptom daily, including cough (37 %), hoarseness (21 %), wheezing (15 %), shortness of breath (10 %), and sore throat (12 %) [16].

Heartburn, the main symptom of GERD, may also occur infrequently (27 %-42 %) in achalasia patients. The mean LES pressure in patients with achalasia who experience heartburn has been reported to be significantly lower than that in patients without heartburn [17]. Weight loss (usually between 5 and 10 kg) is present in most but not all patients. Difficulty belching has also been reported in up to 85 % of the patients, and is due to a defect in relaxation of the upper esophageal sphincter in these patients [1, 18].

## Etiology

The distal esophageal wall and LES are innervated by postganglionic neurons, consisting of excitatory and inhibitory neurons. The excitatory neurons release acetylcholine while the inhibitory neurons release nitric oxide (NO) and vasoactive intestinal polypeptide (VIP), resulting in esophageal and LES contractions and relaxations, respectively [3]. The NO and VIP releasing inhibitory neurons are the target in idiopathic achalasia. Loss of these inhibitory neurons due to either intrinsic or extrinsic causes will result in the manometric consequence of failure of LES relaxation and loss of esophageal peristalsis [3, 4, 19].

Several studies on humans and animals [20, 21] have suggested that extrinsic causes such as lesions located in the central nervous system (CNS) may produce manometric findings of achalasia. Abnormalities of the vagal nerve fibers outside the CNS has also been associated with achalasia; however, extrinsic innervation abnormalities are rare findings in achalasia patients [22–24] and are thus probably not the primary mechanism of the disease. In contrast, intrinsic loss of inhibitory myenteric neurons in both the esophagus and LES has been reported as the most likely contributory factor in the pathophysiology of achalasia. Studies [25–27] have suggested that loss of VIP and NO secreting neurons leads to an imbalance between the excitatory and inhibitory neurons of the myenteric plexus, producing irreversible manometric changes in such patients. Morphologic studies of the esophageal myenteric plexus have also confirmed the loss of myenteric ganglion cells in achalasia [28, 29]. In such studies, loss of ganglion cells was associated with inflammation and in severe cases, the myenteric nerves had been replaced by collagen.

### Genetic

The existence of familial cases suggest that achalasia is an inherited disease [30–33]. Such familial cases have been mostly seen in the pediatric population, between siblings and in a few cases in monozygotic twins [30, 31]. Achalasia has been associated with Allgrove (Triple-A) syndrome, Down’s syndrome, and congenital central hypoventilation syndrome [34].

Mutation of the ALADIN 12q13 gene is a commonly reported cause of achalasia in children. It leads to the development of Allgrove syndrome, also known as Triple-A syndrome, which is an autosomal disease characterized by adrenocorticotropin hormone (ACTH) resistant adrenal insufficiency, achalasia, and alacrima [35]. Furthermore, 77 % of children with Down’s syndrome have gastrointestinal abnormalities with 2 % developing achalasia [36]. Recent studies have also shown multiple genetic mutations such as nitric oxide synthase 1 gene (NOS1), VIP receptor 1, IL23R, IL10 promoter, IL33, and protein tyrosine phosphatase non-receptor 22 (PTPN22) to be associated with the development of achalasia [37–43]. It is proposed that genetic predisposition in such individuals probably increases their susceptibility to acquiring achalasia after exposure to common environmental factors that may play a role in the pathogenesis [2].

### Infection

Several studies have suggested a possible association between viral infections and achalasia [44, 45]. In these studies, various viral antibodies were measured in the serum of patients with achalasia and in normal controls. Measles and varicella zoster virus antibodies were found to be higher among a number of patients with achalasia [3]. Studies using polymerase chain reaction (PCR) demonstrated no evidence of viral products in esophageal tissue of patients with achalasia [46, 47]. More recently, Castaglinolo *et al.* demonstrated HSV-1 reactive immune cells in LES muscles of patients with achalasia [48]. Though HSV-1 may be also found in patients’ without achalasia, the presence of these immune reactive cells directed at the virus suggests HSV-1 may play a role in genetically susceptible individuals. A role for HSV-1 was further demonstrated by Facco *et al.* who demonstrated increased T cell proliferation and T helper-1 type cytokines release in response to HSV-1 antigen from LES muscle specimens harvested during laparoscopic myotomy of 59 patients with idiopathic achalasia [49]. Thus, although the infectious etiology of achalasia remains an unclear matter, there is mounting evidence suggesting an immune-mediated inflammatory disease in which latent HSV-1 infection leads to persistent immune activation and eventual self-destruction of esophageal neurons in genetically susceptible patients [50].

### Autoimmune

Increased prevalence of circulating antibodies against myenteric plexus in some patients with achalasia led to the suggestion of a role for auto-antibodies in the pathogenesis of this disease [51, 52]; however, a recent study by Moses *et al.* [53] suggested that these circulatory antibodies are most likely the result of a nonspecific reaction to the disease process instead of being the cause of the disease. This idea was supported by detection of similar antibodies in patients without achalasia. A recent study did find that patients with achalasia were 3.6× more likely to have autoimmune diseases including Sjogren’s syndrome, uveitis, systemic lupus erythematosus, type I diabetes, and hypothyroidism [54].

Ultra-structural studies [55, 56] of the esophageal tissue of patients with achalasia have also found inflammatory infiltrates around myenteric neurons, while in control groups normal myenteric plexus was found without infiltration. Multiple case–control studies [57–60] have reported a significant association with HLA class II antigens in idiopathic achalasia. A recent study [60] also showed that achalasia patients with the associated HLA allele had a higher prevalence of circulating anti-myenteric autoantibodies, which supports the autoimmune etiology theory [3]. HLA association also suggests immunogenetic predisposition for idiopathic achalasia.

Overall, etiology behind the development of achalasia is likely multifactorial and continues to be highly investigated. Several genes and autoimmune disorders have been associated with achalasia as outlined above. It is proposed that genetic predisposition probably increases the likelihood of triggering autoimmune mechanisms after exposure to viruses or other common environmental factors [61].

## Diagnosis

The diagnosis of idiopathic achalasia is relatively straightforward with a well-documented medical history, radiography, and esophageal motility testing.

### History

In the early stages of the disease, dysphagia may be very subtle and can be misinterpreted as dyspepsia, poor gastric emptying, or stress. The presence of heartburn due to food stasis can add to this confusion. As the disease progresses, difficulty swallowing characteristically occurs with both solids and liquids and is often associated with regurgitation of bland undigested food or saliva [5]. The dysphagia is more to solids than liquids. To ease progression of the food bolus, patients usually modify their eating habits: eating more slowly or using certain maneuvers such as raising the arms or arching the back.

### Esophageal manometry

**Fig. 1:**
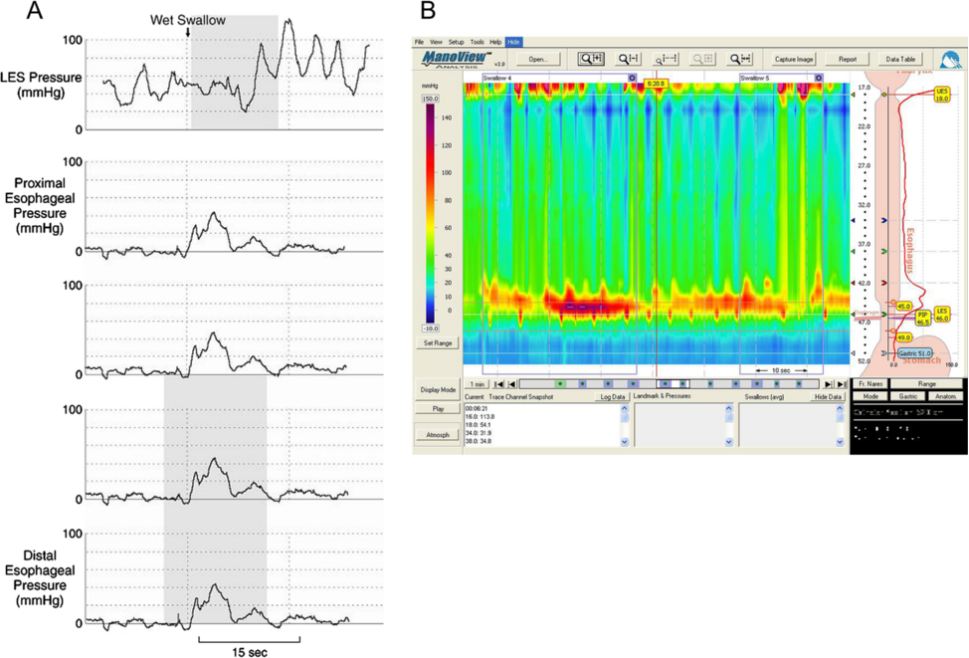
**a**) Conventional water perfused manometric findings of classic achalasia. Isobaric simultaneous esophageal body contractions (lower four tracings) with incomplete LES relaxation (upper most tracing). **b**) High resolution manometry (HRM) findings in achalasia (simultaneous pan esophageal pressurization with incomplete LES relaxation)

Manometry is the gold standard for establishing the diagnosis of achalasia and is essential for the diagnosis regardless of the findings on barium esophagram and esophagogastroduodenoscopy (EGD). The manometric findings of aperistalsis and incomplete LES relaxation is characteristic on conventional manometry. Wet and dry swallows are followed by simultaneous contractions [1]. The amplitude of the contractions is low (10–40 mm Hg) and repetitive in most cases [9] (Fig. 1). The LES displays high pressure at rest and fails to relax, or relaxes only partially with swallowing (Fig. 1). Up to 40 % of the patients with achalasia have normal LES pressure (10–40 mm Hg); however, low pressure LES is not seen in untreated achalasia patients [62].

**Fig. 2:**
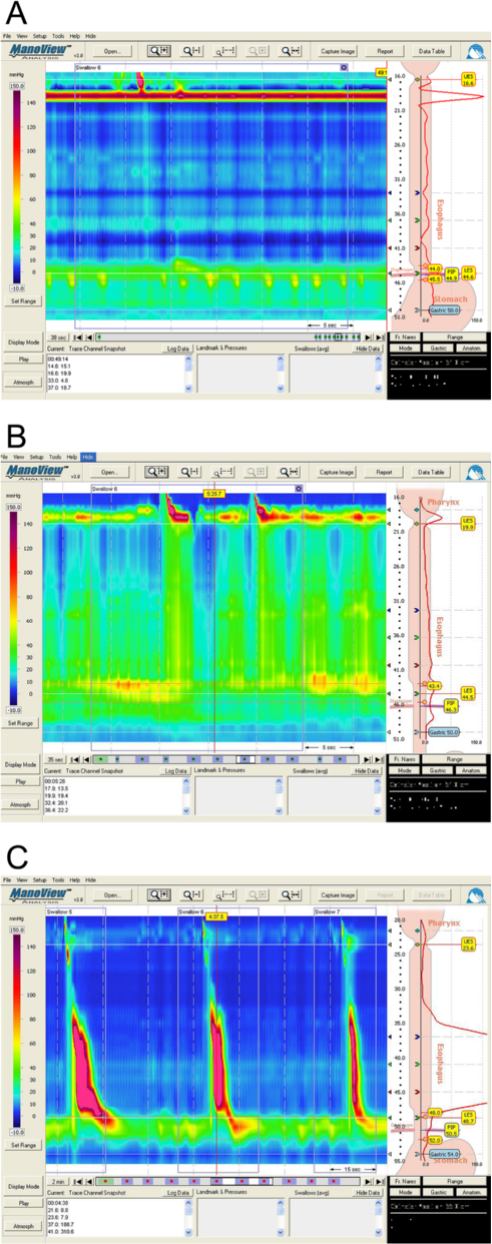
Three sub-types of achalasia on high resolution manometry. **a** Quiescent esophageal body (Type I); **b** isobaric pan-esophageal pressurization (Type II); **c** simultaneous contractions (Type III)

Aperistalsis is defined as lack of propagating esophageal contractile activity and presents with different pressure patterns including quiescent esophageal body (Type I), isobaric pan-esophageal pressurization (Type II), or simultaneous contractions (Type III), and can now be easily identified with high-resolution manometry (HRM) (Fig. 2) [63]. Although both conventional manometry or HRM can be used for diagnosis, new data is emerging to suggest that HRM may have increased sensitivity in diagnosing achalasia compared to conventional manometry techniques [64]. More importantly, new space-time analysis paradigms with HRM that portrays the pressure signal through the esophagus in a seamless dynamic space-time continuum in the form of esophageal pressure topography can help characterize the motor patterns with treatment outcome implications. Based on three retrospective studies, subtype II has the best prognosis, whereas subtype I is somewhat lower and subtype III can be difficult to treat [63, 65, 66].

### Timed barium esophagogram

**Fig. 3:**
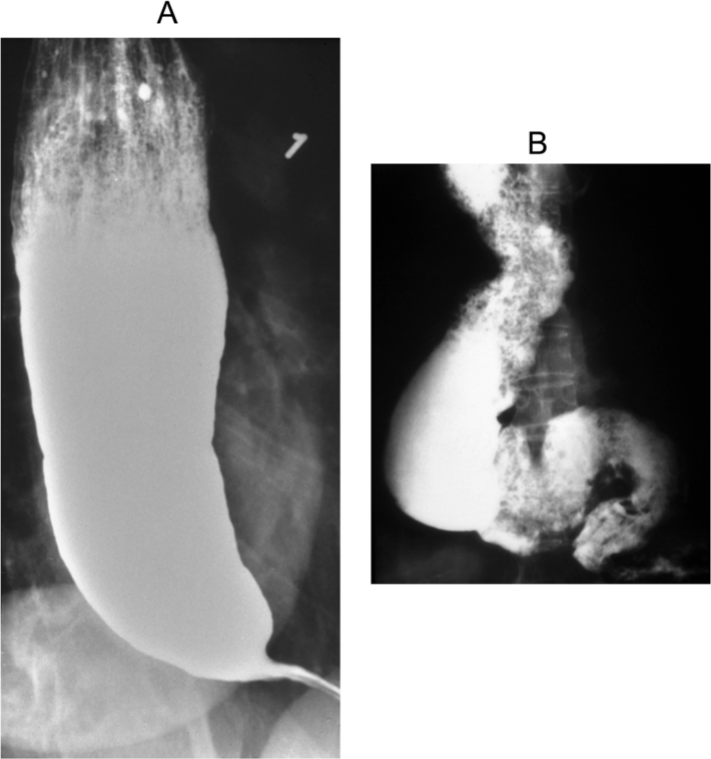
Barium swallow. **a** Dilated esophagus with retained column of barium and “bird’s beaking” suggestive of achalasia. **b** End stage achalasia with retained food, barium and tortuous esophagus

Barium swallow was initially used by Vantrappen et al. [67] in achalasia patients to determine the cause of persistent symptoms after treatment with pneumatic dilation. The characteristics of achalasia in barium esophagogram are the loss of primary peristalsis in the distal two third of the esophagus, and poor emptying with retained food and saliva producing an air-fluid level at the top of the barium column. In chronic stages of the disease, there is a dilated esophagus or sigmoid tortuosity and sometimes, in advanced cases, massive dilatation of the esophageal body that have implications for treatment [1, 5]. The typical finding in achalasia is the presence of smooth tapering of the lower esophagus leading to a closed LES, resembling a bird’s beak (Fig. 3).

**Fig. 4:**
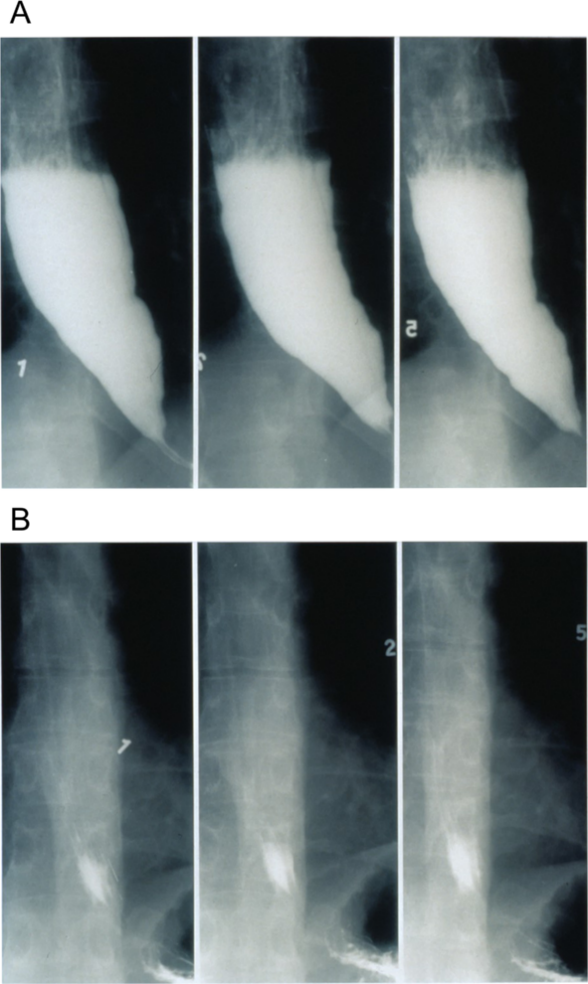
Timed barium swallow before and after pneumatic dilation showing retention of barium in the former and complete emptying post effective therapy in the latter

In 1997 de Oliveira *et al.* [68] described timed barium esophagogram as a simple, noninvasive, and widely available barium technique for evaluating esophageal emptying in patients with achalasia, which can provide objective assessment after therapy as in many patients with achalasia, symptom relief does not always parallel esophageal emptying. The films in this technique are taken at 1, 2 and 5 minutes after the last swallow of barium; the purpose of 2 min film is to assess interim emptying (Fig. 4). The technique is simple to interpret because both radiologists and gastroenterologists can accurately assess emptying. Emptying can be assessed by the height time width of the barium column or a qualitative estimate of emptying. This method can be also used in predicting the success of treatment in patients with achalasia, which will be discussed later [68–71].

### Endoscopy

All patients with suspected achalasia should undergo upper gastrointestinal endoscopy to exclude mechanical obstruction or pseudoachalasia that can mimic achalasia both clinically and manometrically [72, 73]. Pseudoachalasia results from a tumor at the esophagogastric junction, therefore, this area needs to be examined carefully during the procedure [1, 3]. At endoscopy, the esophageal body may look normal, or dilated, atonic and often tortuous. The mucosa looks normal, but sometimes it is thickened or friable with even superficial ulcers secondary to chronic stasis or candida esophagitis. The LES is closed even with insufflations of air, but the endoscope can easily pass this area with gentle pressure. If a tumor is suspected because of rapid progression of symptoms, or the need of excess pressure to open the LES, repeated endoscopy examinations with biopsies and endoscopic ultrasound and CT chest are mandatory. A study by Mittal et al. (2003), also showed that endoscopic ultrasound may be helpful in further evaluation of the LES and ruling out infiltrating tumor if there is stronger resistance on endoscopic evaluation [74].

Although esophageal biopsies are recommended in patients undergoing endoscopic evaluation for dysphagia to assess for eosinophilic esophagitis, biopsies are generally not necessary if the endoscopic findings are characteristic for achalasia [5]. However, it is important to note that it is not uncommon to find an increased number of eosinophils in patients with achalasia secondary to potential stasis inflammation [28, 75], and clinical presentation and classic manometric findings might be necessary to help distinguish the two diagnoses. The information on cancer risk in achalasia is insufficient. There are many studies on this and the great majority of them suggests a significantly increased risk [76]; however, there are currently no recommendations for surveillance of achalasia patients for esophageal cancer.

### Differential diagnosis

A majority of patients are misdiagnosed as having reflux disease given regurgitation. The differential diagnosis of a patient with dysphagia and regurgitation includes GERD, pseudoachalasia (associated with malignancies or secondary achalasia from extrinsic processes such as prior tight fundoplication), iatrogenic achalasia (obstructive procedures for weight loss) and possibly eosinophilic esophagitis. Tumors in the gastric cardia or those infiltrating the myenteric plexus (adenocarcinoma of the gastroesophageal junction, pancreatic, breast, lung, or hepatocellular cancers) should be considered highly in the differential based on findings on EGD and manometry and if the clinical history is significant for acute weight loss [72]. Infection by *Trypanosoma Cruzi,* also known as Chagas’ disease, can also result in achalasia, but these patients often have other features of diffuse enteric myenteric destruction, including megacolon, heart disease, and neurologic disorders [77].

## Management

Despite insight into the pathophysiology of achalasia, the etiology of the disorder remains unknown; thus, it is not surprising that the treatment is entirely palliative. If untreated, the disease course leads to a progressive stasis and dilation of the esophagus, which results in increased risk of aspiration, weight loss, and malnutrition. Current therapeutic options aim to reduce LES pressure, relieve functional obstruction to esophageal transit, and facilitate esophageal emptying. Well-studied treatment options include oral pharmacologic agents, chemical denervation by endoscopic injection of botulinum toxin, pneumatic dilation, and surgical myotomy. More recently investigated endoscopic interventions include self-expanding metallic stents and per-oral endoscopic myotomy (POEM) [78–80]. These methods vary in their level of invasiveness and risk of adverse effects [81, 82].

### Pharmacologic treatment

Oral pharmacologic therapies aim at relaxation of the smooth muscle to lower LES pressure. Calcium channel blockers and nitrates are the two most common agents used [83, 84]. Other less commonly used agents include anti-cholinergics (atropine, dicyclomine, cimetropium, bromide), beta-adrenergic agonists (terbutaline), and theophylline [11]. Calcium channel blockers inhibit calcium entry into the cells, resulting in reduced esophageal muscle contraction and a decrease in LES pressure by 13-49 % [5]. Nifedipine is the most commonly used calcium channel blocker for the treatment of achalasia. It is available in a sublingual formulation with maximum effect seen at 20 to 45 min. Sublingual nifedipine (10-30 mg) should be administered 30–45 min prior to meals and at bedtime [11]. The efficacy of nifedipine largely varies with symptom improvement observed in 0 % to 75 % of patients in clinical trials, but its use is largely limited by side effects reported in up to 30 % of patients [11, 81].

Nitrates work by increasing NO concentration in smooth muscle cells via cyclic GMP. Sublingual isosorbide dinitrate has been shown to reduce LES pressure by 30-65 %, resulting in symptom improvement in 53 to 87 % of patients. The effect of nitrates is more rapid than that of nifedipine, but has a shorter duration; thus, sublingual isosorbide dinitrate (5 mg) is commonly administered only 10 to 15 min before meals [11].

In a study comparing the effect of sublingual nifedipine to sublingual isosorbide dinitrate, both drugs decreased LES pressure, but the effect of nitrate was slightly better than that of nifidipine (65 % *vs.* 49 % respectively) [84] Oral pharmacologic therapy is the least effective treatment option for achalasia and rarely yields satisfactory long-term symptom relief. Additionally, use is limited by side effects such as headache, orthostasis, and pedal edema [85]. Therefore, this treatment modality is reserved for patients who are not candidates for pneumatic dilation or surgery, have failed botulinum toxin injections, or as a bridge to more effective therapy [11].

### Botulinum toxin treatment

Botulinum toxin (BT) was first used in achalasia patients by Pasricha and his colleagues [2, 86, 87]. This toxin is derived from Clostridium botulinum and causes paralysis of voluntary and involuntary muscles by inhibiting the release of acetylcholine from presynaptic vesicles. Local injection of BT results in chemical denervation of the LES; thus, improving esophageal emptying by counterbalancing the selective loss of inhibitory neurons in the myenteric plexus [2, 81]. BT A 80–100 Units are injected through a 5-mm sclerotherapy needle into the LES. Aliquots equaling 20 to 25 U of the toxin are injected into each quadrant of the LES. Injection of BT seems to be simple and safe, without carrying any risk of perforation. Complications are minor and include transient chest pain (16-25 %), reflux symptoms (<5 %), and rare complications such as mediastinitis and allergic reactions related to egg protein [5, 88]. There is also concern that repeated BT injections can induce an inflammatory reaction that may obscure the mucosal-muscular plane and increase surgical complications during future surgical myotomy [82, 89–91].

Although initial symptom relief is observed in >75 % of patients, the therapeutic effect wears off, and approximately 50 % of patients will require repeat injections at 6 to 24 month intervals, or additional treatment with pneumatic dilation (PD) or myotomy [1, 2, 87, 92]. Table 1 reflects the symptom response rate as well as percent of LES pressure drop after treatment with BT over a period of 12 months in most valuable studies. Only a few studies are available on the long-term efficacy of BT. Our initial randomized trial found a one year success rate of 32 % in achalasia patients treated with BT [2]. Annese *et al.* [93] reported a success rate of 68 % at 24 months after receiving repeated BT injection, while Pasricha *et al.* [94] found a 30 % efficacy rate after a mean follow-up of 2 years. Post-treatment evaluations have revealed that neither pre-treatment LES pressure, amplitude of esophageal contractions, nor duration of illness could be used to predict the outcome of BT injection. Instead, young age and male gender were found to adversely affect the outcome [94, 95]. Symptom relief was found to last up to 1 to 2 years with a single injection in the elderly [96, 97]. Overall, BT is recommended to be most effective in elderly patients, in whom dilation or surgery represent a high risk.

**Table 1:** Effect of botulinum toxin on achalasia

Study	Method	Number of patients enrolled	% LES pressure decreased post treatment	Remission rate at 1 months	Remission rate at 6 months	Remission rate at 12 months
Pasricha *et al.* [87]	Randomized control trial	21	33 %	90 %	44 %	___
Fishman *et al.* [92]	Prospective study	60	___	70 %	___	36 %
Gordon *et al.* [123]	Prospective study	16	___	75 %	48 %	___
Vaezi *et al.* [2]	Randomized trial	24	1 %	60 %	50 %	32 %
Annese *et al.* [116]	Randomized trial	16	49 %	100 %	___	12.5 %
Pasricha *et al.* [94]	Prospective study	31	45 %	90 %	64 %	___
Martinek *et al.* [124]	Prospective cohort study	49	65 %	93 %	___	41 %
Zaninotto *et al.* [125]	Randomized controlled trial	40	___	___	66 %	34 %

### Pneumatic dilation

Pneumatic dilation (PD) is the most effective non-surgical treatment option for patients with achalasia [9]. It uses air to dilate the esophageal lumen and disrupt the circular muscle fibers of the LES [1]. The most commonly used balloon is the Rigiflex dilator. Rigiflex balloons come in three different diameters (3.0, 3.5, and 4.0 cm). Initial dilation with a 3.0 cm balloon is recommended for most patients. The pressure required is usually 8–15 psi of air held for 15–60 s. The number of dilation sessions depends on recurrence of symptoms and there are scoring systems available such as the Eckardt score system, which can be used to define patient’s response to PD [98]. Patients undergo a post-procedure gastrograffin study followed by barium esophagram to rule out esophageal perforation [5].

Cumulatively, dilation with 3.0, 3.5, and 4.0 cm balloon diameters results in good to excellent symptomatic relief in 74 %, 86 %, and 90 % of treated patients, respectively, with an average follow-up of 1.6 years [11]. Patients often require repeat intervention over time due to decreased remission rates. In a study of 106 patients, Vela *et al.* reported the success rate of single PD as 62 % at 6 months and 28 % at 6 years, compared to 90 % at 6 months and 44 % at 6 years with serial dilation [99]. In a prospective study, Eckardt *et al.* treated 54 patients with PD and reported an overall 5-year remission rate of 40 % and a 10-year remission rate of 36 % [95]. Other studies have shown good to excellent symptom improvement in 50-89 % of patients over a mean follow-up of 4 years [11, 100, 101].

Predictors of favorable clinical response to PD include older age (>45 years), female gender, narrow esophagus, LES pressure after dilation of <10 mmHg, increased emptying on post-treatment timed barium esophagram, and type II pattern on HRM. In younger males, it is recommended that the PD employing the 3.5 cm balloon or surgical myotomy may be the best initial approach [5, 99]. Pneumatic dilation is well-tolerated with a rare but serious complication of esophageal perforation in approximately 2 % of procedures [11]. Due to the risk of esophageal perforation, patients being considered for PD must be surgical candidates in case of perforation.

Our recent studies [69, 70, 100, 102] suggest timed barium esophagogram as a better predictor of treatment success after PD. We have found that in almost 70 % of the patients, the height of the barium column at 5 min post-therapy correlates with symptom improvement (concordant group), while in others esophageal emptying was poor despite reports of excellent symptom relief (discordant group). Nearly all patients in discordant group failed the treatment within 1 year after treatment, while 77 % of the concordant group were still in symptom remission after 6 years of follow-up [69]. Therefore, it is suggested that the timed barium esophagogram not only assesses treatment shortly after therapy, it can also predict the poor response to the treatment if the patient has retained barium post-pneumatic dilation.

### Surgical myotomy

Surgical management of achalasia involves performing a Heller myotomy (HM), combined with a fundoplication to prevent reflux. Surgical myotomy was originally performed via thoracotomy with good to excellent results in 60-94 % of patients followed for 1–36 years [11]. This intervention evolved to be performed with a laparotomy approach, then a thoracoscopic approach, and finally via laparoscopy that has fallen in favor due to superior visualization of the gastroesophageal junction, the ability to add an anti-reflux procedure, decreased morbidity, shorter hospital stay, and faster recovery [103].

A cumulative good to excellent clinical response rate of 94 % has been reported for laparoscopic myotomy over a short period of time. Studies on long-term outcome of myotomy are summarized in Table 2. The major disadvantages of myotomy are incomplete myotomy and the possibility of significant GERD. Table 2 also shows the rate of developing GERD after myotomy in the most valuable studies reported. In a double-blind randomized trial, Richards *et al.* reported abnormal acid exposure on pH monitoring in 47 % of patients without an anti-reflux procedure compared to 9 % of patients who had a Dor fundoplication [104]. While a concomitant anti-reflux procedure is recommended [105], the type of fundoplication (Dor vs. posterior Toupet) remains controversial with recent meta-analysis showing significantly higher recurrence rate of clinical regurgitation and pathological acid reflux in the Dor fundoplication group [106].

**Table 2:** Long-term result of laparoscopic myotomy with fundoplications

Study	Method	Method of surgery	Number of patients enrolled	Length of follow-up	Good to excellent response	GERD^a^ complication
Bessell *et al.* [126]	Prospective	Laparoscopic HM^b^	167	5 years	77 %	Not mentioned
Vella *et al.* [99],	Retrospectivecohort	88 % Laparoscopic and 12 % open HM	73	6 years	57 %	36 %
Dang *et al.* [127]	Retrospective	81 % Laparoscopic and 9 % open HM	22	3 years	76 %	Not mentioned
Raiser *et al.* [128]	Retrospective	Laparoscopic or thoracoscopic HM	35	1-4 years	97 %	Not mentioned
Hunt *et al.* [129]	Retrospective	Laparoscopic HM	70	2.9 years	81 %	4.5 %
Frantzides *et al.* [130]	Retrospective	Laparoscopic HM	53	3 years	92 %	9 %
Zaninotto *et al.* [131]	Prospective	Laparoscopic HM	100	2 y	92 %	7 %

^a^GERD Gastroesophageal reflux disease; ^b^HM Heller myotomy

Rebecchi *et al.* reported similar long-term reflux control in patients who received a Heller myotomy plus floppy-Nissen versus Dor fundoplication; however, those who underwent Nissen fundoplication experienced more recurrence of dysphagia [107]. Subsequently, multiple recent randomized controlled trials comparing Heller myotomy in conjunction with a Dor versus Toupet fundoplication showed significant improvement in both dysphagia and regurgitation symptoms regardless of the type of partial fundoplication with dramatic improvements in Eckardt scores [108, 109]. Patients undergoing Toupet fundoplication did have significantly better relative improvements in the EORTC QLQ-OES18 (functional scale), but otherwise no differences between the two anti-reflux repairs were noted [109]. Thus, the optimum fundoplication procedure after HM for achalasia remains controversial, but given the likelihood of reflux symptoms after myotomy despite added fundoplication, proton pump inhibitor (PPI) therapy may be indicated in those who complain of heartburn [5].

Possible treatment options after a failed myotomy include PD or a repeat surgical myotomy. A study of untreated achalasia patients and patients with failed myotomy reports no increased risk of perforation with performing PD after Heller myotomy. However, this study also indicates that despite lower LES pressure, patients undergoing PD after failed myotomy do not do as well as untreated cases [110]. Finally, laparoscopic surgery used to be performed only on patients who relapsed after graded PD; however, PD and surgical myotomy are now offered as initial therapy for patients who are at low surgical risk. Studies show best outcomes after PD in patients older than 40 years, women, those with narrow esophageal diameter, and those with a type II pattern on HRM [63, 69, 99, 111, 112], while surgery may be indicated as the first line therapy for patients with tortuous esophagus, esophageal diverticula, or previous surgery on the gastroesophageal junction.

### Per-oral endoscopic myotomy

Per-oral endoscopic myotomy (POEM) is an endoscopic approach to esophagomyotomy that was first reported in porcine models by Pasricha *et al.* [79] and then in humans by Inoue *et al.* [113]. Since its introduction in humans in 2010, this novel approach has been increasingly used at centers worldwide. POEM involves an esophageal mucosal incision (approximately 10 cm proximal to esophagogastric junction) followed by creation of a submucosal tunnel and dissection of the muscle fibers beginning at 3 cm distal to the mucosal entry site and extending 2 cm into the cardia [114]. Since it is essential to prevent an inadequate myotomy, extension of the submucosal tunnel beyond the LES into the cardia is confirmed via a retroflexed view with visualization of the blue dye (used in the submucosal injection). The incision is subsequently closed with hemostatic clips or endoscopic suturing [114]. This procedure is technically demanding and requires a certain level of training and expertise. However, treatment success has been reported as high as 90 % with significant decreases in LES pressure, decreased Eckardt scores, and improved quality of life measurements with low complication rates [115]. The main complication is GERD that has been reported to occur in approximately 12 % of patients. Other rare potential complications include delayed bleeding, pneumomediastinum, pneumothorax, pneumoperitoneum, and mucosal flap perforation [114]. POEM appears to be a promising intervention; however, current data is limited by small study numbers and short-term follow-up.

### Comparison of the procedures

Several randomized trials suggest that pneumatic dilation is more effective than botulinum toxin [2, 116, 117]. Our study, which was one of the largest studies comparing the outcome of two therapies, suggested that both therapies are effective at 1 month, but PD results in significantly better symptom improvement at 12 months compared with BT (70 % *vs.* 32 % respectively) [2]. These findings indicate that botulinum toxin is inferior to pneumatic dilation for sustained symptom relief [118]. A study on cost-effectiveness of treatments also suggests that in the long-term, PD is a more cost-effective treatment for achalasia compared to BT [119].

Comparison of PD and HM also shows that there is no difference in the early outcome of these treatments, and the success rate of both methods decreases over time (90 % *vs.* 89 % respectively at 6 months, to 44 % *vs.* 56 % at 6 years) [99]. Studies also suggest that laparoscopic myotomy is not a cost-effective therapy as the initial cost is too high [119]. However, HM has improved cost-effectiveness and the difference between the two treatment modalities decreases if the durability of HM is >10 years given the likelihood of necessary repeated PD treatments [120]. Laparoscopic HM is an effective treatment modality in patients with achalasia who have failed to respond to PD, as the 10-year remission rate in these patients following myotomy is shown to be 77 % compared to 72 % and 45 % in patients “successfully” treated with a single PD and patients undergoing several dilations respectively [121]. Overall, HM is the more durable treatment for achalasia, but PD is more cost-effective. A recent randomized trial comparing HM to PD showed equivalent treatment outcome after two years of follow up in 201 patients with achalasia [111] . Subsequently, a comparative study between POEM and HM showed that both interventions resulted in similar symptom and physiology improvement, but POEM resulted in shorter operative times and shorter hospitalizations [122].

**Fig. 5:**
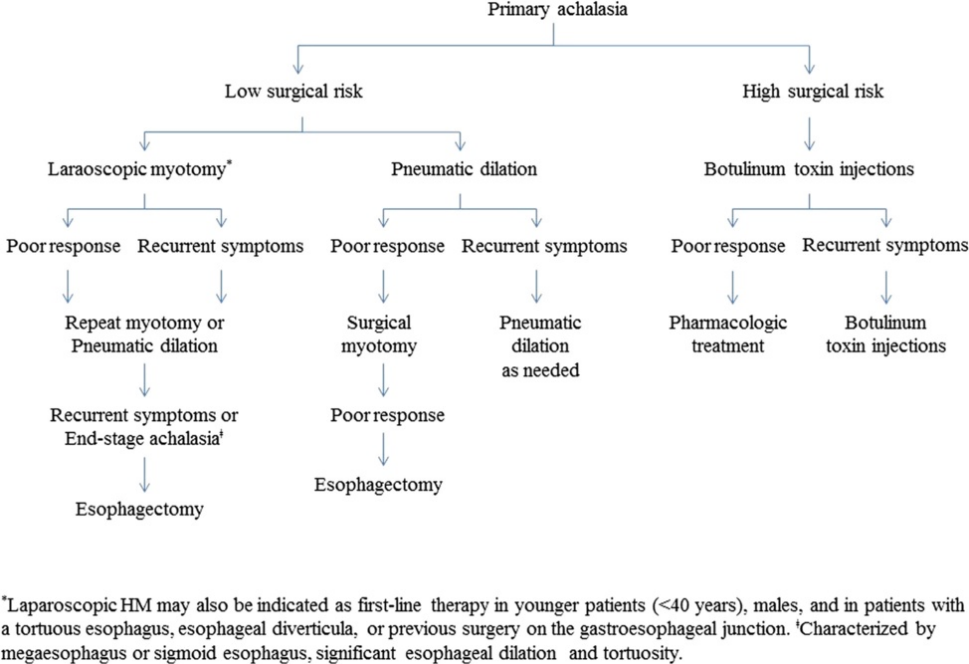
Management algorithm for patients with achalasia

A proposed algorithm for the management of patients with achalasia is depicted in Fig. 5. The choice of initial therapy should be guided by patients’ age, gender, preference, and local institutional expertise. Surgical myotomy and PD remain the key treatment options for patients. When deciding on myotomy vs PD, it is important to consider the complications, durability, and cost-effectiveness, as well as the experience of the surgeons and gastroenterologists. Botulinum toxin therapy is recommended for patients who are not surgical candidates or are high-risk, and pharmacologic therapy is reserved for patients who cannot undergo definitive treatment or have failed botulinum toxin injections.

## Conclusions

Achalasia is a motor disorder of the esophagus characterized by dysphagia, regurgitation, and chest pain. Although it cannot be permanently cured, excellent palliation is available in over 90 % of patients. As a result of the advances in pneumatic dilation and laparoscopic Heller myotomy, most patients with achalasia can now choose between these two treatments. The injection of botulinum toxin endoscopically into the LES is usually reserved for elderly, or patients who are not candidates for pneumatic dilation or surgery. In patients unresponsive to graded pneumatic dilation, esophageal myotomy via the laparoscopic method should be performed.
